# Multiple Histone Methyl and Acetyltransferase Complex Components Bind the *HLA-DRA* Gene

**DOI:** 10.1371/journal.pone.0037554

**Published:** 2012-05-31

**Authors:** Nancy M. Choi, Jeremy M. Boss

**Affiliations:** Department of Microbiology and Immunology, Emory University School of Medicine, Atlanta, Georgia, United States of America; Texas A&M University, United States of America

## Abstract

Major histocompatibility complex class II (MHC-II) genes are fundamental components that contribute to adaptive immune responses. While characterization of the chromatin features at the core promoter region of these genes has been studied, the scope of histone modifications and the modifying factors responsible for activation of these genes are less well defined. Using the MHC-II gene *HLA-DRA* as a model, the extent and distribution of major histone modifications associated with active expression were defined in interferon-γ induced epithelial cells, B cells, and B-cell mutants for *MHC-II* expression. With active transcription, nucleosome density around the proximal regulatory region was diminished and histone acetylation and methylation modifications were distributed throughout the gene in distinct patterns that were dependent on the modification examined. Irrespective of the location, the majority of these modifications were dependent on the binding of either the X-box binding factor RFX or the class II transactivator (CIITA) to the proximal regulatory region. Importantly, once established, the modifications were stable through multiple cell divisions after the activating stimulus was removed, suggesting that activation of this system resulted in an epigenetic state. A dual crosslinking chromatin immunoprecipitation method was used to detect histone modifying protein components that interacted across the gene. Components of the MLL methyltransferase and GCN5 acetyltransferase complexes were identified. Some MLL complex components were found to be CIITA independent, including MLL1, ASH2L and RbBP5. Likewise, GCN5 containing acetyltransferase complex components belonging to the ATAC and STAGA complexes were also identified. These results suggest that multiple complexes are either used or are assembled as the gene is activated for expression. Together the results define and illustrate a complex network of histone modifying proteins and multisubunit complexes participating in *MHC-II* transcription.

## Introduction

Antigen presentation is a paramount step in achieving adaptive immunity, where the major histocompatibility class II complex (MHC-II) proteins play a central role. The significance of MHC-II complexes is best illustrated in cases of bare lymphocyte syndrome (BLS) as patients that are unable to express MHC-II suffer from various bacterial and viral infections and usually do not survive beyond childhood [1]. MHC-II proteins display antigenic peptides sampled from the endocytic compartments of the cell onto the cell surface; these peptides typically originate from extracellular pathogens but can include self, viral, or cancer-cell derived peptides. Recognition of MHC-II-peptide complexes by CD4 T cells triggers the expansion and differentiation of these T cells, leading to a host of antigen-specific immune responses [2]. Proper expression of MHC-II proteins both spatially and temporally is critical, as aberrant expression can lead to an insufficient immune response or autoimmunity [3].

*MHC-II* genes are expressed constitutively in professional antigen presenting cells and thymic epithelial cells, and can also be induced in most other cell types following treatment with interferon-γ (IFN-γ) [4], [5]. Cell-type dependent expression is largely controlled by regulation of a limiting transcription factor, the class II transactivator (CIITA) [6]. *MHC-II* genes share a highly conserved proximal upstream promoter region called the WXY box, where the factors RFXAP/B/5, CREB, and NF-Y bind directly, forming a scaffold that is recognized by CIITA [7], [8]. This unique DNA-protein structure is collectively called the MHC-II enhanceosome [9]. The RFX proteins and CIITA are essential for *MHC-II* expression, as genetic deficiencies in these proteins leads to a MHC-II null phenotype and BLS [1]. Located approximately 2.4 kb upstream of the transcription start site resides another WXY element that is fully functional. Initially described as a locus control element [10] and later termed *XL4* for conserved homology with the WXY sequence [11], *XL4* binds RFX and CIITA. Although the exact mechanism is unknown, it was proposed that *XL4* regulates *HLA-DRA* through a looping mechanism [11]. No other distal regulatory elements were reported to regulate *HLA-DRA*. CIITA also interacts with a multitude of coactivating factors and general transcription factors, which are recruited to the promoter to fine-tune the expression of *MHC-II* genes (reviewed in [12]). It has been previously shown by chromatin immunoprecipitation (ChIP) that multiple histone acetylation modifications and active methylation marks increased with constitutive and induced *MHC-II* expression at the proximal conserved promoter regions of some *MHC-II* genes, suggesting a role for these marks in regulation of this system.

Lysine acetylation modifications were the first histone modifications to be assigned an activating role in gene transcription, and were initially described to be localized at promoter regions, as well as enhancer and insulator elements of most genes [13], [14]. A well characterized histone lysine acetyltransferase (KAT) CREB-binding protein (CBP) is recruited to *MHC-II* genes through interactions with the N terminus of CIITA and possibly with phosphorylated CREB that is bound at the promoter [15], [16], [17]. CBP and its homologue p300 are capable of acetylating all core histones but have a preference for histone H4K5 and K8 when tested with mononucleosomes in vitro but can also acetylate (ac) histone H3K14 and K18 [18], [19]. Another group of well-studied histone acetyltransferases of the GNAT family, PCAF (p300/CBP-associated factor) and GCN5 (general control nonderepressible 5), have a preference for histone H3 with specificity for H3K14 and H4K8 in vitro. PCAF and GCN5 have an expanded specificity for histone H3K9, K14, K18, K23, and K27 in vivo, depending on the complex of proteins with which they are incorporated [19], [20], [21]. GCN5 and PCAF are also recruited to the promoter of *MHC-II* genes [22]. PCAF has been shown to acetylate CIITA, altering its nuclear localization as a mechanism to regulate *MHC-II* transcription [23]. It has been shown previously that there is an accumulation of multiple acetylation marks on histone H3K9, K18, K27, and K14, as well as H4K5 and K8 in wild-type B cells and in non-myeloid cells with IFN-γ induction [24], [25]. The level of H3K18ac was affected when recruitment of CBP was reduced at the DRA promoter due to knockdown of the proteasome component Sug1 [26]. However, the same treatments did not affect histone H3K9ac, suggesting a requirement for an independent (or redundant) KAT complex to catalyze this modification [26]. While KATs may have the capability to acetylate diverse residues, they have a very restricted specificity in vivo, and by determining the various modifications that occur, it may be possible to identify the factors responsible for the modifications and their regulatory roles in transcription. Additionally, a number of complexes containing the same KATs have been described, with each complex likely having specificity for individual genes regulating distinct pathways. In the case of *MHC-II* genes, it is not known which complexes are actually bound.

Methylation of histones can cause varied outcomes depending on the modified residue and number of methyl groups [27]. The most well studied active transcription methylation marks are those of histone H3K4, where the three different levels of H3K4 methylation (me) have been shown to be associated with different regulatory functions and areas of the genome. In reference to the transcriptional start site (TSS), histone H3K4me3 (trimethylation) is associated with actively transcribed genes and focused to a narrow region immediately around the TSS. Histone H3K4me2 (dimethylation) has a broader deposition over the TSS that trails into the coding region and has been regarded as the ‘open’ chromatin mark, suggesting that the region is accessible but not necessarily transcribed. H3K4me1 (monomethylation) has attracted attention as the mark of enhancers and other regulatory elements, but is also found within the coding region of transcribed genes [28]. Several groups have shown H3K4me2/3 (di and tri), H3R17me2, K36me2/3, and K79me2, all marks associated with transcriptionally active genes, to be induced with *HLA-DRA* expression, as well as loss of silencing marks H3K9me2/3 and H3K27me3 [11], [25], [29], [30], [31].

The first identified H3K4 methylating enzyme was Set1 (SET domain containing 1) in yeast as a subunit of a multipartite complex called COMPASS (Complex Proteins Associated with Set1) [32], [33]. Set1 and its human counterpart MLL1 (mixed myeloid leukemia) are both homologs of *Drosophila* trithorax, well known for its histone H3K4 methylating activity and role in the positive regulation of homeobox genes during development [34]. Proteins of the MLL core complex have been shown to bind and promote H3K4 methylation at the *HLA-DRA* promoter [35], and intriguingly, interaction between MLL and PML (promyelocytic leukemia) has been shown to prolong the dimethylation state of H3K4 and maintain transcriptional memory by localization to PML nuclear bodies [36]. Collectively, these data support the possibility of an MLL/COMPASS type histone lysine methyltransferase (KMT) complex having a role in *MHC-II* regulation.

While we have a basic understanding of some of the key promoter proximal histone modifications and some of the factors involved, we do not know the overall distribution of these modifications and factors, their stability or complexity, nor do we fully understand their dependence on CIITA. To further define the role of the major histone modifications associated with transcriptional activation to this gene, we assessed the levels of various modifications across the *HLA-DRA* MHC-II gene in constitutive and inducible cell systems. Using a dual crosslinking ChIP protocol to increase the radius of crosslinking and interaction capture, the observed histone modifications were correlated with the binding of specific coactivator/histone modifying complexes that are constitutively present or recruited to the locus upon induction with IFN-γ. The results showed that a multitude of factors, including those that make up the MLL complex and complexes containing GCN5 and PCAF, are involved at the *HLA-DRA* gene and are likely associated with regulating its expression.

## Materials and Methods

### Cell Lines

The human Burkitt’s lymphoma cell line, Raji, was purchased from the American Type Culture Collection (Manassas, VA) [37], RJ2.2.5 cells are a CIITA-deficient cell line derived from Raji by γ-irradiation mutagenesis [6], [38], and SJO cells are an RFX5-deficient cell line isolated from a BLS patient [39], [40], [41]. RJ2.2.5 and SJO are MHC-II negative. SJO cells were generously provided by Dr. J. Gorski (Wisconsin Blood Center), and RJ2.2.5 cells were provided by Dr. R. Acolla (University of Insurbia, Italy). The above cell lines were cultured in RPMI supplemented with 5% fetal bovine serum, 5% bovine calf serum, 100 U/ml penicillin, and 100 µg/ml streptomycin. A431, a human epithelial cell line, was obtained from ATCC and cultured in Dulbecco’s Modified Eagle’s Medium with 10% fetal bovine serum, 100 U/ml penicillin, and 100 µg/ml streptomycin. A431 cells were treated with 500 U/ml IFN-γ (PeproTech, Inc., Rocky Hill, NJ) for 24 hrs when indicated to induce the expression of *CIITA* and *HLA-DRA*. In some experiments, IFN-γ treated cells were washed with growth media and cultured in IFN-γ-free media for the indicated time.

### qPCR and Primers

For all real-time quantitative PCR reactions, Bio-Rad iCycler instruments (Bio-Rad Laboratories, Inc., Hercules, CA) with an iQ optical module were used to measure the amount of SYBR incorporated amplicons. DNA oligonucleotides used for primers listed in Table 1 were purchased from Integrated DNA Technologies, Inc. (Coralville, IA) and diluted to a final concentration of 100 nM for PCR reactions. All primers were tested by agarose gel electrophoresis to ensure that they formed single amplicon products of the correct size and optimized for Tm by temperature gradient real-time PCR followed by a melt curve analysis. Standard curves of sonicated genomic DNA were used to quantify the amount of starting material for every PCR reaction.

**Table 1:** DNA oligos used in real-time PCR reactions.

Primer	forward	reverse
CIITA*	5′-CTGAAGGATGTGGAAGACCTGGGAAAGC	5′-GTCCCCGATCTTGTTCTCACTC
HLA-DRA*	GAGTTTGATGCTCCAAGCCCTCTCCCA	CAGAGGCCCCCTGCGTTCTGCTGCAAT
GAPDH*	CCATGGGGAAGGTGAAGGTCGGAGTC	GGTGGTGCAGGCATTGCTGATG
XL4	CAGAGAAAGGGAACTGAAAGTCATTT	TTATGACACTGTTTAGTCCTAGAACACTGA
−2000	CAACAACTTGGATTGAAGATGC	AGGTAAAGAGTCAGGAGAATGG
−600	ATGAGATACAATGCCAGCCATCC	ACAGTTGGAGAGTTTGCGTAAGG
−300	TGTCCCTTACGCAAACTCTCC	ACACAAGATACTCCGTTCATTGG
WXY	GATCTCTTGTGTCCTGGACCCTTTGCAAGAACCCT	CCCAATTACTCTTTGGCCAATCAGAAAAATATTTTG
+300	GGACGATAGACTACGAAGCATTGG	TGACTTACTTCAGTTTGTGGTGAGG
+600	AGCCCTGTTCTTATCTGAATACATG	GCCTTCCCTCCCCTTTTCC
+1500	CTCCGTCTCAAACAACCAAACC	ACCAACACCAAGGGAATAATGAAC
+3500	TTCCGCAAGTTCCACTATCTCC	CGAGTTTCACACAAGCATCATAGG
+5800	AGGTAAAGAGTCAGGAGAATGG	ATGATACAGCCAAGATGAAACC

* Primers used for Reverse Transcription PCR reactions.

### mRNA Extraction and RT-PCR

IFN-γ treated and untreated A431 cells were grown until they were 80% confluent and RNA was prepared from cell pellets using the RNeasy kit (Qiagen, Venlo, Netherlands) according to manufacturer’s recommendations. 2 µg of total mRNA was used in reverse transcription reactions using Superscript II (Life Technologies Corp., Carlsbad, CA) with oligo dT and random hexamer primers (Life Technologies Corp., Carlsbad CA). Transcript specific primers (Table 1) were tested to produce single amplicon products prior to the qPCR reaction. Real-time PCR data were normalized to *GAPDH* mRNA expression using the ΔΔCt method [42]. All experiments were conducted at least three times from independent cell cultures and statistical significance was determined by Student’s t-test.

### Chromatin Immunoprecipitation (ChIP)

**Figure 1:**
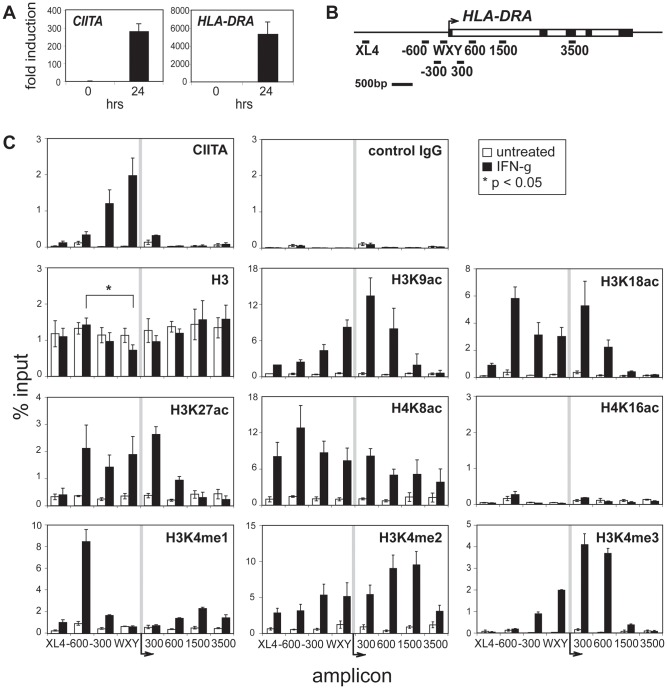
IFN-γ treatment induced the deposition of active histone modifications throughout the *HLA-DRA* gene. (A) RNA levels for *CIITA* and *HLA-DRA* were measured by qRT-PCR. Transcript levels for A431 cells are shown before and after treatment with 500 U/ml of IFN-γ for 24 hrs. The results of three independent experiments were averaged and plotted with standard error. (B) A schematic of the *HLA-DRA* gene and open reading frame is illustrated with exons (black boxes) and introns (clear) indicated. The bars below the gene represent the relative position of the PCR amplicons used. (C) ChIP and qPCR were conducted on untreated control (clear) or 24 hrs IFN-γ treated (black) A431 cells using the indicated antisera and amplicons described in B. The data are presented as an average of the percent input value derived from three to four biological replicates and error bars represent standard error. For unmodified histone H3, the asterisk (*) indicates a Student’s t-test value of p<0.05 in comparing an upstream region (−600) and the WXY box region with IFN-γ treatment.

**Figure 2:**
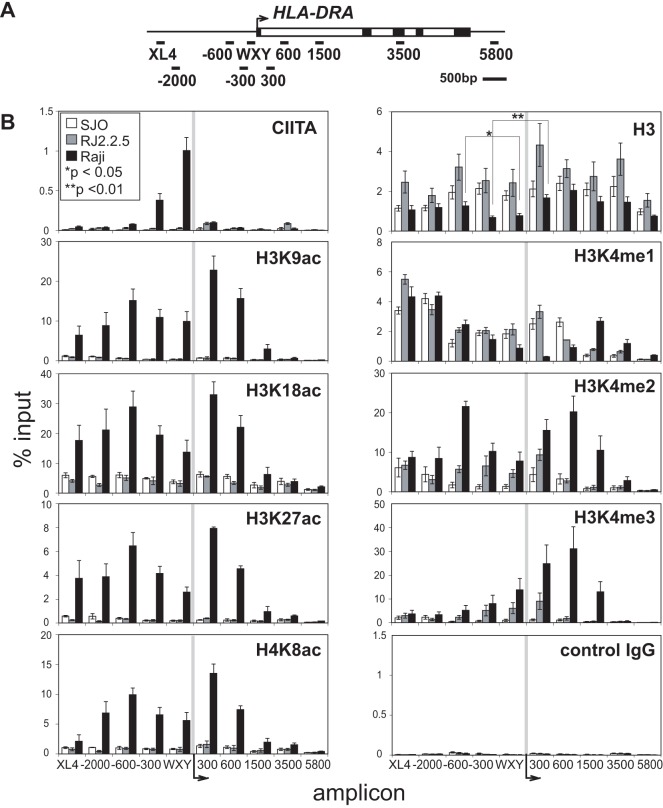
*MHC-II* expressing B cells have active histone modifications distributed across the *HLA-DRA* gene. (A) The *HLA-DRA* gene region and additional PCR amplicons (−2000 and +5800) used in this and subsequent experiments are depicted. (B) Using the indicated antibodies, the distribution of unmodified H3, CIITA, and modified histones at the *HLA-DRA* gene was analyzed by ChIP-qPCR for the amplicons described in A in *MHC-II* expressing (Raji, black) and non-expressing (RJ2.2.5, CIITA-deficient, grey; SJO, RFX5-deficient, clear) B cell lines. The data are plotted as the average of the percent input values from three biological replicates and error bars represent standard error. In the unmodified histone H3 panel, an asterisk (*) indicates a Student’s t-test value of p<0.05 comparing the −600 and −300/WXY region, and double asterisks (**) indicate a t-test value of p<0.01 between +300 and −300/WXY in Raji cells.

For ChIP of histones, a conventional procedure was used as described [24]. For suspension cells, 4×10^7^ cells were crosslinked by 1% formaldehyde for 10 min. Adherent cells were plated the previous day onto two 15 cm plates such that they would be 80% confluent 16–24 hrs later when formaldehyde is applied. Following crosslinking, chromatin was sonicated until the majority of it was reduced to 200–600 bp fragments using the Bioruptor water bath sonicator (Diagenode, Denville, NJ) and the amount of chromatin DNA was measured using the Bio-Rad VersaFluor Fluorometer (Bio-Rad Laboratories, Inc., Hercules, CA). 10 µg of chromatin and 2.5 µg of antibodies were used per ChIP assay. 10% of each IP was used for each subsequent PCR reaction. Antisera were purchased from various manufacturers as follows: anti-H3, H3K18ac, H3K4me1 (Abcam plc., Cambridge, United Kingdom), H3K9ac, H3K27ac, H4K16ac, H3K4me2, H3K4me3 (Millipore Corporation, Billerica, MA). A polyclonal anti-T cell receptor (TCR) antibody was used as a non-specific negative control antibody in the histone ChIP experiments. All data were presented as the percent of the input chromatin. Figures 1 and 2 were presented as unnormalized data, whereas the same data presented in Figures S1 and S2 were normalized to the levels of unmodified histone H3 at each amplicon.

**Figure 3:**
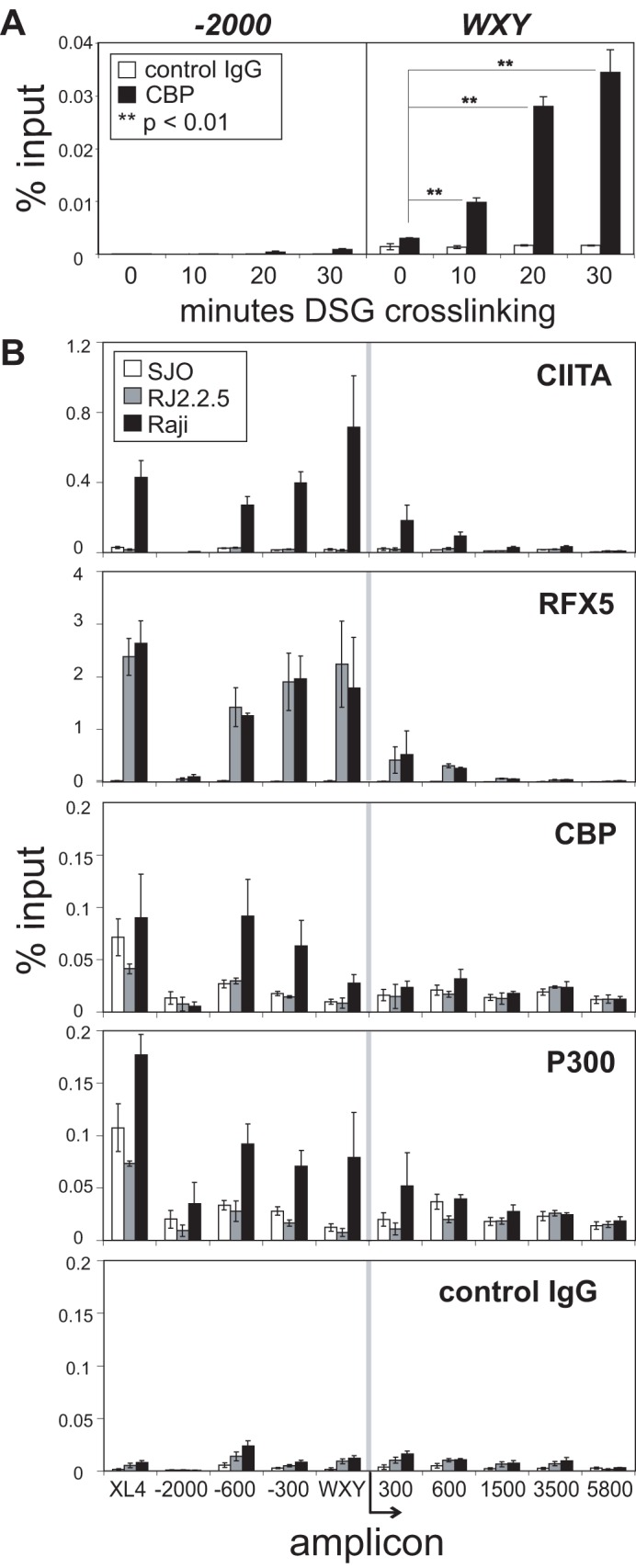
Dual crosslinking enhances the pulldown efficiency of coactivators CBP and p300. (A) ChIP-qPCR was performed for CBP at the *HLA-DRA* promoter region (WXY) and a negative control region (−2000) after Raji cells were treated with disuccinimidyl glutarate (DSG) for the indicated time together with the conventional formaldehyde crosslinking. Clear columns indicate the percent input values for control IgG pulldown and black columns represent those for CBP. (B) ChIP-qPCR was performed for the indicated factors in Raji (black), RJ2.2.5 (grey; CIITA-deficient), and SJO (clear; RFX5-deficient) cells. Amplicons tested by qPCR were as illustrated in Figure 2A. The values plotted are an average of three biological replicates and error bars represent standard error. The double asterisks (**) in A denote a Student’s t-test value of p<0.01 when compared to 0 min DSG crosslinked values.

For non-histone proteins, a secondary crosslinker was used in addition to formaldehyde. Disuccinimidyl glutarate [43] (DSG, Proteochem, Denver, CO) was applied to cells at 2 mM in Sodium phosphate buffer (pH 8.0) for indicated times at room temperature. 1% formaldehyde was added to cells for the last 10 min of crosslinking with the DSG. All successive steps were identical to conventional ChIP. Except for the time course experiment in Figure 3A, all DSG treatments were carried out for 20 minutes. 30 µg of chromatin was immunoprecipitated with 5 µg of anti-CBP, p300, GCN5, PCAF, MLL1 (Santa Cruz Biotechnology, Inc., Santa Cruz, CA), ASH2L, RbBP5 (Bethyl Laboratories, Inc., Montgomery, TX), ATAC2, YEATS2, NC2-β, TRRAP, TADA1L, WDR5 (Abcam plc., Cambridge, United Kingdom), and DPY-30 (Abnova, Taipei, Taiwan). Anti-CIITA antibodies were prepared as previously described [44]. ADA2a and ADA2b antibodies were a generous gift from Dr. R.G. Roeder at The Rockefeller University [45]. Unimmunized control rabbit IgG (Millipore Corporation, Billerica, MA) was used as a non-specific negative control antibody for all transcription factor/complex ChIP assays. The mean and standard error from these assays were provided as percent input of the chromatin added. For all ChIP assays, the Student’s t-test was applied to determine statistical significance and all experiments were repeated at least three times with separate cell culture preparations.

### siRNA Treatment and Immunoblotting

Raji cells were split the previous day to allow the cells to grow in log phase and collected the next day for nucleofection. ON-TARGET plus SMARTpool siRNA for non-targeting control, GCN5, MLL1, and WDR5 were purchased from Dharmacon (Thermo Fisher Scientific Inc., Waltham, MA) and resuspended in nuclease free water. Using the Amaxa Nucleofection kit V (Lonza, Basel, Switzerland) 1×10^6^ cells were transfected with each indicated pool of siRNA [100 nM] and cultured for 72 hrs. These siRNA transfected cells were either collected for immunoblot, RNA analysis, or ChIP. For immunoblotting, cells were washed with PBS and lysed with RIPA buffer (25 mM Tris pH 7.6, 150 mM NaCl, 1% NP-40, 1% sodium deoxycholate, 0.1% SDS) to generate whole cell lysates. Protein concentrations were measured using the Bio-Rad protein assay reagent (Bio-Rad Laboratories, Inc., Hercules, CA) and equal amounts of lysate protein were resolved on a polyacrylamide gel and transferred to a PVDF membrane. Membranes were blocked with 5% non-fat milk dissolved in TBS-Tween20 (0.01%) solution then probed with each indicated antiserum. Antibodies to beta-actin (Santa Cruz Biotechnology, Inc., Santa Cruz, CA) were used to portray sample loading. HRP conjugated anti-mouse and anti-goat were from Sigma-Aldrich Co. (St. Louis, MO), and anti-rabbit was from Rockland Immunochemicals Inc. (Gilbertsville, PA), respectively. Chemiluminescence signal was recorded on BioMax XAR film (Kodak, Rochester, NY) and band intensities were measured using ImageQuant TL program (GE healthcare, Piscataway, NJ).

## Results

### IFN-γ Treatment Leads to Increased Active Histone Marks at the *HLA-DRA* Locus

Histone modifications and the factors that place these marks are responsible for the regulation of the chromatin state and may influence the expression of a gene. Identification of potential factor candidates can be obtained from an understanding of the presence and distribution of the local chromatin modifications across the locus. To generate this information, a series of ChIP assays were conducted on cells that were induced for *MHC-II* gene expression, as well as those that constitutively expressed the genes. The *HLA-DRA* gene was used as the model *MHC-II* gene because it is monomorphic and expressed at high levels. The A431 epithelial cell line is negative for both *CIITA* and *MHC-II* gene expression. Treatment with IFN-γ induced expression of both *CIITA* and *HLA-DRA* genes ∼300 and ∼5,000 fold, respectively (Figure 1A).

To assess the distribution of histone modifications across the *HLA-DRA* locus, a set of amplicons were designed along the length of the *HLA-DRA* gene stretching from −2,500 to +3,500 bp (Figure 1B), which included the upstream regulatory element (here termed *XL4*) [10], [11], to a region far downstream of the TSS. Amplicons for −300 and the major regulatory region, WXY box, are immediately adjacent to each other (Figure 1B). The −300 amplicon is where the first nucleosome 5′ of the nucleosome free region has been reported to be positioned [46]. ChIP analyses of A431 cells (−/+ IFN-γ) were performed using anti-CIITA and various histone posttranslational modification specific antibodies that are associated with active transcription (Figure 1C). CIITA occupancy appeared only after IFN-γ induction and showed a sharp peak at the WXY and −300 regions, which confirms that it was indeed recruited to the *HLA-DRA* gene [8], [24] in our assay, and also that the resolution of the experiment was ∼300 bp. Analysis of unmodified histone H3 across the region showed minor differences across the gene in the uninduced state. However, following IFN-γ induction the distribution of nucleosomes was reduced at the WXY region compared to an upstream region (−600) as previously described [46], suggesting that this region is more accessible in the active state.

Histone lysine acetylation marks were examined for H3K9, K18, K27, and H4 acetylation marks K8 and K16 (Figure 1C). In the uninduced state, very low levels of these modifications were observed, irrespective of the position across the gene. Following IFN-γ induction, a broad distribution of the acetylation marks appeared. Histone H3K18 and K27 acetylation showed relatively even distributions around the TSS and WXY box regions; whereas H3K9 and H4K8 displayed different patterns with H4K8 acetylation levels being higher upstream and H3K9 acetylation levels peaking at regions downstream from the TSS. Histone acetylation levels of H3K14, K23, and H4K5, K12, and K16 were also measured following IFN-γ treatment. Very low to no levels of these modifications were detected at the control or induced time point (data not shown) as represented by H4K16ac in Figure 1C. Thus these data point to H3K9 and H3K18 as the dominant H3, and H4K8 as the dominant H4 acetylation modifications associated with IFN-γ induced *HLA-DRA* gene expression. The levels of histone modifications in Figure 1C are plotted irrespective of histone H3 concentrations, whereas Figure S1 shows the same data normalized to the levels of histone H3 ChIP at each amplicon. Only slight differences in distribution over the WXY and −300 amplicons can be observed in comparing the two figures; which reflect the change in nucleosome density following IFN-γ treatment.

As H3K4 methylation is associated with active transcription, the three degrees of methylation were observed following IFN-γ treatment. H3K4me1 levels were significantly induced at all regions except the WXY box and +300. Surprisingly, the −600 amplicon showed high levels of the H3K4me1 modification. There is no reported regulatory activity for this region at this time. The finding of this modification may suggest a novel regulatory element at this location. The mark for histone H3K4me2 showed a broad distribution, but was clearly shifted downstream from the TSS. H3K4me3, a mark of active transcription was more sharply positioned around the TSS with a peak downstream of the TSS. Significant levels of H3K4me1 and me2 modifications were also found at the upstream *XL4* element with IFN-γ induction. It is important to point out that the baseline levels of all the above modifications are typically very low, suggesting that CIITA occupancy is required for these modifications.

### Active Histone Marks are CIITA and RFX5 Dependent in B Cells at *HLA-DRA*

To determine if a constitutively expressing system would have a similar histone modification distribution, a commonly used Burkitt’s lymphoma B cell line, Raji, was examined. Raji cells express high levels of *CIITA* and all *MHC-II* genes, including *HLA-DRA*. To determine whether the above histone modifications were dependent on the presence of CIITA or RFX5, ChIP assays using CIITA- (RJ2.2.5) and RFX5- (SJO) deficient cell lines were compared to Raji. RJ2.2.5 cells were derived from Raji cells by γ-irradiation and SJO cells were B cells established from a bare lymphocyte syndrome patient [6], [38]. The region of analysis was expanded to +5800 with an additional amplicon at −2000. This analysis encompasses the entire open reading frame of *HLA-DRA* (Figure 2A).

In Raji cells, a high and tight peak of CIITA binding was observed at the WXY region as expected (Figure 2B, [24]). Nucleosome density measured by histone H3 presence was variable between the three cells lines. In Raji cells, there is a clear reduction in nucleosomes at the −300/WXY region compared to its surrounding areas; whereas the nucleosome density in RJ2.2.5 or SJO cells did not show this preferential depletion at the proximal promoter region (Figure 2B). This agrees with the IFN-γ induction data presented above, suggesting that only in the active state does the chromatin structure of the proximal promoter region become accessible.

The presence of the activation marks observed above was examined in the three cell lines. Universally, all four acetylation modifications were lower or absent in RJ2.2.5 and SJO cells compared to Raji. As RJ2.2.5 cells do not make a functional CIITA, and SJO cells do not bind CIITA due to a lack of RFX binding, these data demonstrate that CIITA is required for these modifications. In Raji cells, histone H3K9 and K18 acetylation values were extremely high and were broadly distributed extending upstream of the gene and only slightly into the open reading frame. H3K27 and H4K8 levels were also high and showed a similar distribution. In contrast to the IFN-γ pattern, *XL4* showed higher levels of the histone H3 acetylation marks in B cells, with the exception of H4K8ac, which had shown a relatively high level following induction by IFN-γ. As above, Figure 2 is plotted irrespective of the nucleosome density; whereas Figure S2 is plotted with respect to the histone H3 levels for each amplicon. While there are no major differences, histone modifications associated with the WXY and −300 regions are increased in Raji cells when normalized to the lower levels of nucleosomes over those sequences.

Histone H3K4 methylation showed distinct distributions among the wild-type and mutant B-cell lines. Monomethylation was strongest at the upstream *XL4* site and the surrounding region in all cell lines, which is indicative of this region functioning as an enhancer-like element [14]. However, in Raji cells, monomethylation was markedly decreased at regions approaching the TSS, disappearing at +300, and reappearing at +1500 bp downstream of the TSS. In contrast monomethylation was higher at the +300 region in RJ2.2.5 and SJO cells and positioned evenly throughout the −600 to +600 regions. No pronounced peak at −600 was observed in any of the cells. Histone H3K4me2 was broadly distributed in Raji cells and lower at all regions in RJ2.2.5 and SJO cells. The levels of H3K4me2 in SJO cells were significantly lower than RJ2.2.5 at the WXY box, suggesting that the lack of RFX binding to the region may be responsible for this additional loss of this histone modification. Histone H3K4me3, as expected showed sharp and high levels at regions close to the TSS in Raji cells, with its highest level at +600. In RJ2.2.5, low levels of H3K4me3 were observed close to the TSS. In contrast, no H3K4me3 was observed in SJO cells, again pointing to the role of RFX5 in assembly of the factors at the WXY box region [47].

### Dual Crosslinking ChIP Increases Pulldown Efficiency

The spacer length of formaldehyde is limited to 2 Å, a distance that allows for the ChIP assay to be optimal for DNA binding proteins or their tightly interacting protein partners [48]. However, detection of coactivators, chromatin modifiers, or factors that interact at a greater distance from the DNA may not be as efficient under the standard conditions. To enhance the pulldown efficiency of our ChIP assays for indirect DNA-protein interactions, a dual crosslinking ChIP procedure was optimized using disuccinimidyl glutarate (DSG), which has a crosslinking arm of 7.7 Å [43]. Using this dual crosslinking assay, detection of CBP, a known coactivator that interacts with CIITA and binds to the promoter [15], [30], was significantly increased at the WXY box (Figure 3A). The increase in CBP detection was time dependent, and importantly, CBP binding was absent at a negative control region (−2,000) even at the longest time treatment of DSG. This demonstrates that there is no increase in non-specific binding due to the additional crosslinking step, while increasing the pulldown efficiency more than 9 and 11-fold when cells were DSG crosslinked for 20 or 30 min, respectively, compared to cells only crosslinked with formaldehyde. Therefore, the DSG dual crosslinking procedure was employed in subsequent ChIP assays to examine coactivator proteins interactions with the *HLA-DRA* gene.

Additional controls were carried out across the region to assure that backgrounds levels were not increased at each amplicon, and previously reported coactivators could be captured (Figure 3B). Using all three cell lines, CIITA, RFX5, CBP, p300, and control IgG antisera were used in the dual crosslinking ChIP assays. The results showed a slight broadening of CIITA and RFX5 binding at the WXY/−300 regions. RJ2.2.5 showed RFX5 binding as before [10] and SJO did not bind either factor. The dual crosslinking procedure did however produce higher levels of these factors at *XL4*. Control IgG levels were low at each amplicon. CBP and p300 binding was also captured at the WXY through −600 amplicons in Raji cells but not in RJ2.2.5 or SJO suggesting that their recruitment was dependent on CIITA. This role of CIITA recruiting these factors to the promoter region was reported previously [15], [16], [22], [30]. However, significant recruitment of CBP and p300 were found at *XL4* in all three cell lines, suggesting that their recruitment to this region is independent of RFX5 and CIITA.

### The MLL Histone Methyltransferase Complex Components are Enriched at Surrounding Regions

**Figure 4:**
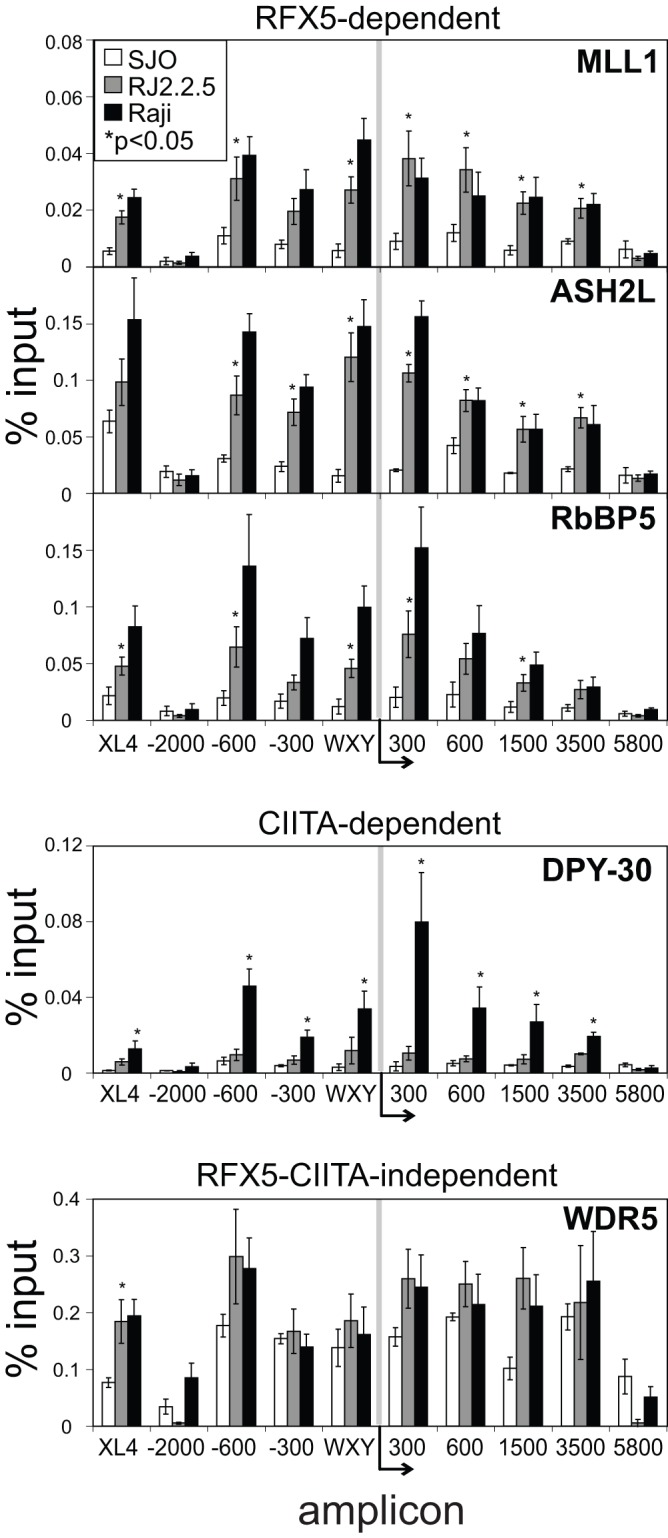
Histone methyltransferase MLL1 core complex proteins are recruited to *HLA-DRA in B cells.* MLL core complex proteins, MLL1, WDR5, ASH2L, RbBP5, and DPY-30 were assessed for their binding across the *HLA-DRA* gene in Raji (black), RJ2.2.5 (CIITA-deficient, grey), and SJO cells (RFX5-deficient, clear) by qPCR after DSG-ChIP. The negative control IgG ChIP pulldowns conducted concurrently with these experiments are shown in Figure 3B. The data represent the average of the percent input values from the pulldown of each indicated protein at each amplicon as indicated in Figure 2A. The values are an average of 3–5 biological replicates and the error bars represent the standard error. For RFX5-dependent factors, the asterisks represent Student’s t-test values p<0.05 of statistical significance for comparisons between RJ2.2.5 and SJO. For CIITA-dependent factors, the asterisks represent Student’s t-test values p<0.05 between Raji and RJ2.2.5 cells.

The MLL histone methyltransferase core complex consists of MLL, WDR5, ASH2L, RbBP5, and DPY-30 [47]. Together these proteins catalyze the addition of a methyl group to H3K4, producing mono-, di-, and trimethylated H3K4 in vitro and in vivo [47], [49]. As there were high levels of all three methylation states observed at different regions across the *HLA-DRA* gene, ChIP assays were conducted on Raji, RJ2.2.5, and SJO chromatin preparations to determine if these MLL complex core proteins were present (Figure 4). It was expected that, because these factors are part of a core complex that binding to the *HLA-DRA* locus, would simply reflect the transcriptional activation state of the locus. This was not the case. Whereas all five proteins were bound across the locus in Raji cells, MLL1, ASH2L, and RbBP5 were mostly dependent on the presence of RFX5, while showing slight dependency on CIITA. DPY-30 was unique in that its binding was completely RFX5 and CIITA dependent as binding was not detected in either SJO or RJ2.2.5. WDR5 recruitment was completely independent of RFX5 or CIITA due to the fact that its level of occupancy was unchanged between the three cell types. RFX and CIITA independent binding of some of these subunits may explain why lower but significant levels of H3K4me2 were observed in RJ2.2.5 cells when compared to Raji cells.

**Figure 5:**
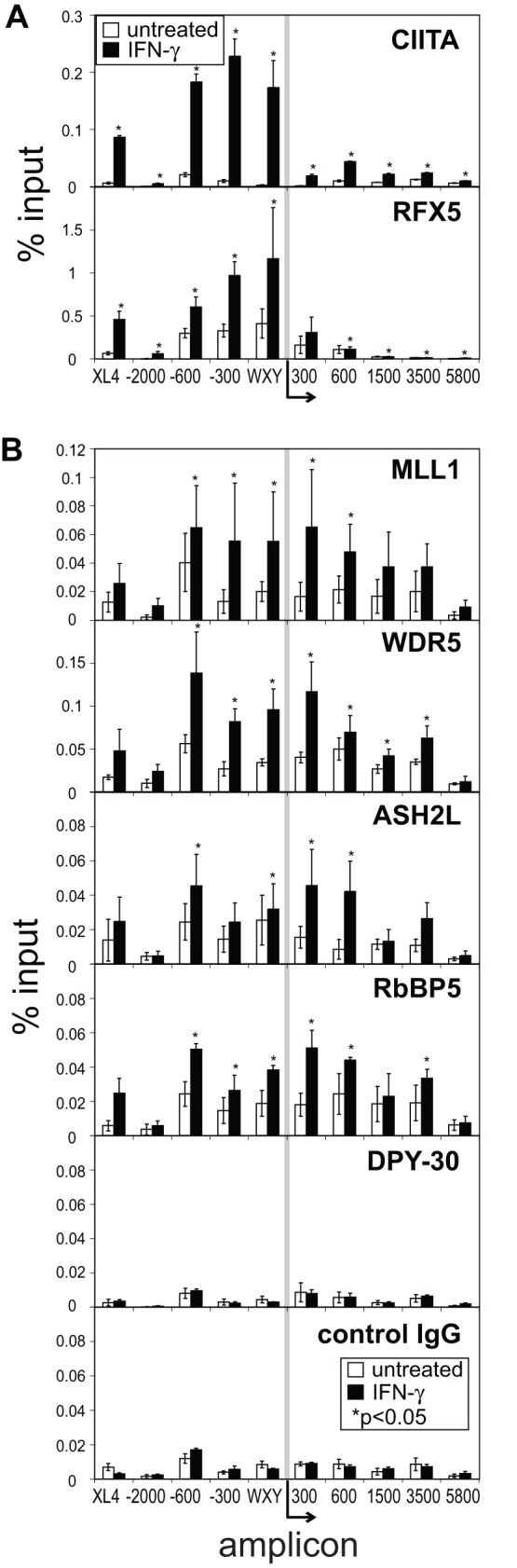
MLL core complex proteins are recruited in IFN-γ treated cells . (A) Human epithelial A431 cells were treated with IFN-γ for 24 hrs and tested for the binding of CIITA and RFX5 to verify the experimental procedure in the IFN-γ inducible system. (B) MLL core complex proteins were tested for binding before and after IFN-γ treatment in A431 cells. The data presented represent the average percent input value at each amplicon (Figure 2A) after DSG-ChIP pulldown of three biological replicates from untreated (clear) and IFN-γ treated (black) cells. The error bars represent the standard error. Asterisks represent Student’s t-test values p<0.05 of the IFN-γ treated cells compared with background IgG controls for that amplicon.

IFN-γ induced A431 cells were also tested to determine the binding of the above factors. CIITA and RFX5 ChIPs were conducted initially to test the efficiency of dual crosslinking pulldown in the IFN-γ induced cells. CIITA binding was present only in the IFN-γ treated cells. The levels of pulldown achieved in the inducible system was ∼4 fold lower than in B cells at the WXY box (Figure 5A). While there are low levels of RFX5 binding in the resting A431 cells, they are induced more than 2 fold with IFN-γ treatment. This has been observed previously, and is due to stabilization of the DNA binding components by CIITA [50], [51]. Therefore, an increased level of coactivator binding can be due to both the increased recruitment of RFX or CIITA. In uninduced cells, only WDR5 displayed significant binding above the background IgG control (Figure 5B). Following IFN-γ treatment, MLL1, WDR5, ASH2L and RbBP5 were associated with sequences surrounding the TSS and at *XL4*. Surprisingly, DPY-30 was not observed at the locus under any of the conditions tested. Together, these results suggest that the MLL histone methyltransferase components are being recruited to the *HLA-DRA* locus most significantly at the promoter, but also within the body of the gene and regulatory regions.

### Histone Acetyltransferase Complexes Containing GCN5 and PCAF are Present throughout the Coding Region and at Upstream Regulatory Elements

**Figure 6:**
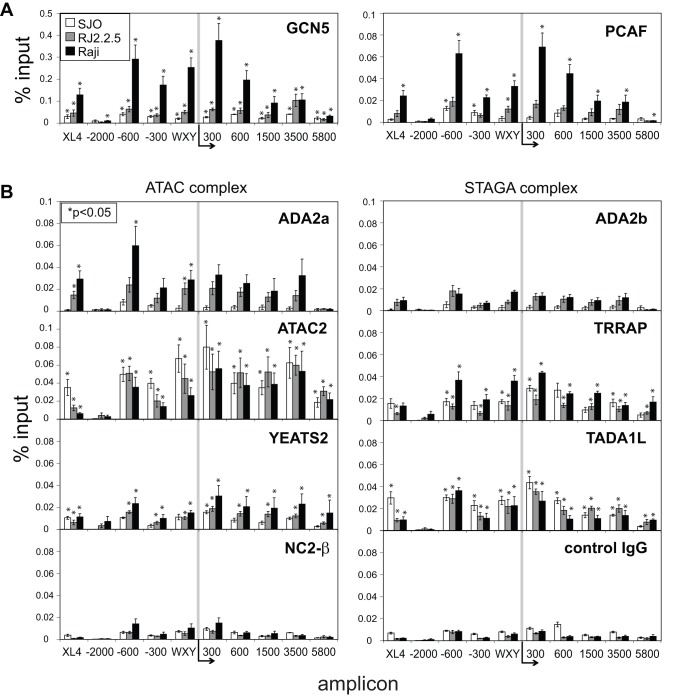
GCN5 and PCAF containing histone acetyltransferases ATAC and STAGA are recruited to *HLA-DRA.* (A) Dual crosslinking ChIP-qPCRs for histone acetyltransferases GCN5, and PCAF were conducted for regions across *HLA-DRA* in Raji (black), RJ2.2.5 (CIITA null, grey), and SJO (RFX5 null, clear) cells as above. (B) Binding of the ATAC (ADA2a, ATAC2, YEATS2, NC2-β) and STAGA (ADA2b, TRRAP, TADA1L) complex subunits was determined as in A. The average of percent input values from the DSG-ChIP for each indicated protein is shown with the values averaged from 3–5 biological replicates. The asterisks indicate a Student’s t-test value of p<0.05 at each amplicon when compared to the control IgG values for each cell line.

The lysine acetyltransferases (KAT) CBP and GCN5 have been shown previously to bind the *HLA-DRA* promoter [22], [26], [30]. CBP, p300, and PCAF have been shown to interact directly with CIITA [15], [16], [23], [52]. To examine the breadth at which these KATs interact across the locus and to correlate that with the histone acetylation data, ChIP assays for these factors were carried out in the three B cell lines described above. Thus, to determine whether CIITA or RFX5 was indeed important in recruiting each of these factors to the *HLA-DRA* locus the binding of the above KATs in Raji, RJ2.2.5, and SJO cells were compared. There were no major differences in the protein expression levels of these KATs between the three cell lines as determined by immunoblot (Figure S3). At the WXY box region, the presence of CIITA was critical for CBP, p300, GCN5, and PCAF (Figures 3B and 6A), illustrating the role CIITA plays in the recruitment of these KATs to the locus. The binding of these KATs to *XL4* was also CIITA dependent. In agreement with the histone acetylation data (Figure 2B), a high level of GCN5 and PCAF were observed at the −600 region. Moderate levels of GCN5 and PCAF, but not CBP and p300, were associated within the coding region.

Recent studies in humans have defined two distinct GCN5/PCAF containing complexes, termed STAGA and ATAC that diverged from their shared common ancestor, the yeast SAGA complex [53], [54]. To determine which of these complexes might participate in *HLA-DRA* gene expression, ChIP assays for a number of key subunits of each complex were conducted (Figure 6B). For the ATAC complex, ADA2a, ATAC2, YEATS2, and NC2-β were examined. The overall binding levels of these components were low and may be due to antibody affinity. The binding of ADA2a and YEATS2 displayed some level of binding across the locus in Raji and RJ2.2.5 cells, suggesting that their recruitment was dependent on RFX5 and CIITA. ATAC2 displayed statistically significant binding in all three cell types, suggesting that its recruitment was independent of RFX5 and CIITA. Binding of NC2-β was not detected for any of the regions. For STAGA complexes, ADA2b, TRRAP, and TADA1L were examined. ADA2b showed no statistical significance over background IgG in binding in any of the cell types (Figure 6B). TRRAP and TADA1L were bound in all three cell types, with TRRAP displaying significantly higher levels in Raji over RJ2.2.5 at several of the loci examined. Thus, while the presence of ADA2a versus ADA2b would suggest that the ATAC complex and not STAGA was bound, components of each of these complexes could be found associated with the *HLA-DRA* gene.

A similar analysis for the IFN-γ treated A431 cells was also conducted and is presented in Figure S4. Albeit at lower levels, GCN5 was found at significant levels from −600 through +3500 over background in both untreated and IFN-γ treated cells. A statistically significant increase in GCN5 was observed surrounding the promoter region (−300 through +300). Of the other factors examined, only ATAC2, TRRAP, and TADA1L showed low but statistically significant binding and this binding was not dependent on IFN-γ.

### siRNA Knockdowns of GCN5 and MLL do not Affect Transcription or Histone Modification Levels

**Figure 7:**
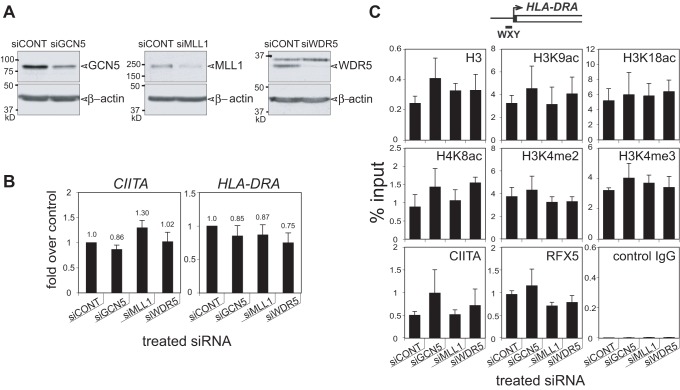
GCN5, MLL1, and WDR5 knockdown does not affect *HLA-DRA* expression or histone modifications. siRNA SMARTpools representing a control pool or the indicated gene were transfected into Raji cells using nucleofection. (A) At three days post transfection western blots for the indicated factor were performed along with β-actin control. (B) mRNA from similar cultures was analyzed by qRT-PCR for *CIITA* and *HLA-DRA* transcripts. The data from four biological replicates was plotted over the siRNA control transfection. (C) Chromatin was isolated from the indicated siRNA transfected cells at 3 days post transfection and analyzed for the presence of the indicated histone modifications at the WXY box region of the *HLA-DRA* gene. The results of three biological replicates were averaged and plotted with respect to the input chromatin. Error bars in B and C represent the standard error.

To examine whether GCN5 or MLL play a non-redundant and critical role in the regulation of *HLA-DRA* gene expression, siRNA knockdowns of these proteins and WDR5 were conducted in Raji cells. Compared to a control non-targeting Dharmacon SMARTpool, siRNA SMARTpools to GCN5, MLL1, and WDR5 were able to reduce the levels of their respective proteins between 50 and 80% (Figure 7A). Analysis of *CIITA* and *HLA-*DRA gene mRNA levels at 3 days post transfection (Figure 7B) or 5 days post transfection with a second siRNA transfection at 48 hrs after the initial transfection (data not shown) showed no change in expression of either gene. Consistent with this result was the finding that histone H3K9ac, H3K18ac, H4K8ac, H3K4me2, H3K4me3 levels were not altered and CIITA binding was not affected (Figure 7C). A similar set of experiments conducted with A431 fibroblasts −/+ IFN-γ treatment and the siRNAs also did not provide a clear dependency on these factors for expression (data not shown). Thus, although these factors are readily detectable at the *HLA-DRA* gene, their roles are not essential to expression, there are functionally redundant factors, or once the gene is activated, the transcriptional complexes are stable.

### Histone Modifications are Stably Present after Removal of IFN-γ

**Figure 8:**
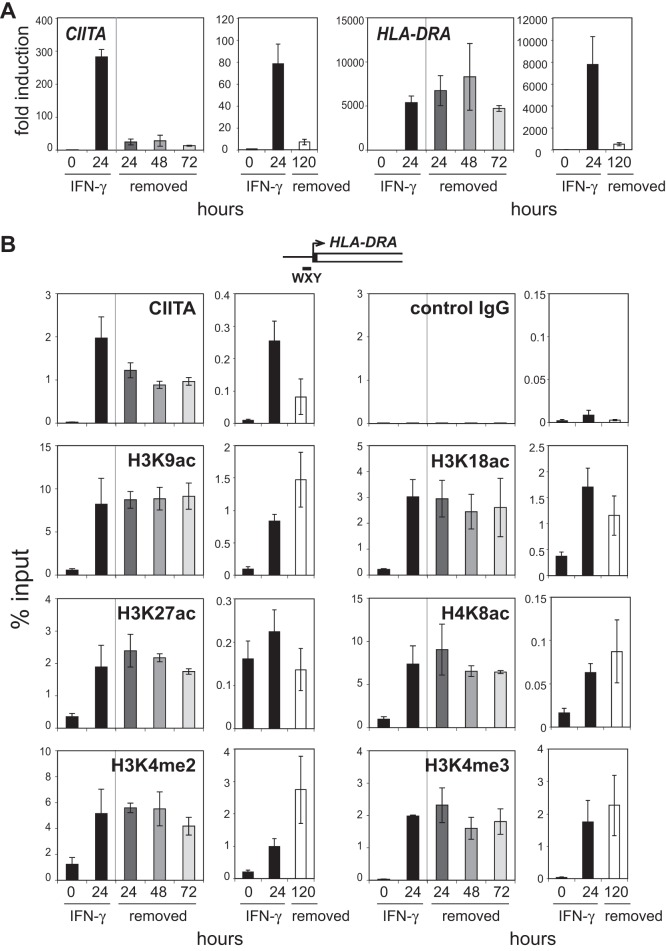
CIITA and modified histones are stably bound at the *HLA-DRA* promoter after IFN-γ removal. (A) mRNA levels of *CIITA* and *HLA-DRA* were measured by qRT-PCR after A431 cells were treated with IFN-γ for 0 or 24 hrs (black bars), and after IFN-γ treated cells were washed and supplied with IFN-γ free media and cultured for an additional 24 to 72 hrs (grey bars). An additional but separate set of experiments were carried out for 120 hours (clear) and are shown. The results of three independent experiments were averaged and plotted with standard error. (B) ChIP for CIITA and each indicated histone modification, as well as a negative control IgG were conducted after A431 cells were treated as in A. ChIP assays were analyzed at the WXY box region. The qRT-PCR and ChIP values for the 0 and 24 hr IFN-γ treated samples in the left panels of each set are the same as those shown in Figure 1 as the data were generated from the same set of experiments and chromatin preparations, and are provided again only for comparison purposes.

To address the possibility that once induced, the histone modifications associated with *HLA-DRA* expression are stable, A431 cells were treated with IFN-γ for 24 hrs as in above experiments, then washed and supplied with culture media free of IFN-γ and cultured for an additional 24, 48, or 72 hrs. During the course of these experiments the cells double at least once per day. Within 24 hrs of IFN-γ removal, *CIITA* mRNA levels were substantially lower in these cells, whereas *HLA-DRA* mRNA levels did not significantly change up to 72 hrs (Figure 8A). CIITA binding at the *HLA-DRA* promoter was assessed by ChIP to determine whether IFN-γ removal also diminished recruitment of CIITA (Figure 8B). After removal of IFN-γ, CIITA levels at the promoter gradually decreased to ∼49% of the initial induction. When histone modification levels were examined, none of the modifications tested showed a significant reduction up to the 72 hr time point. An additional set of experiments was carried out for 5 days after IFN-γ removal. At this time point, *CIITA* and *HLA-DRA* mRNA levels had fallen to ∼7 and 400 fold over pre-stimulation levels, respectively. CIITA binding to the *HLA-DRA* WXY region was reduced by ∼68%. Intriguingly, the histone modifications were for the most part unchanged even at these later time points (Figure 8B). These results illustrate the stability of CIITA binding at the *MHC-II* promoters, as well as the stability of the histone modifications.

## Discussion

*MHC-II* genes, as a fundamental constituent of adaptive immunity, are highly regulated at the transcriptional level. While many essential cis-regulatory elements and transcription factors have been determined, only a few coactivating transcription factors have been identified. In this report, our efforts were focused on determining the location and distribution of active histone modifications associated with *MHC-II* gene transcription, and on identifying histone modifying coactivating factors that were recruited to the *HLA-DRA* gene, which served as a model MHC-II gene. Along with histone modifications, the relative nucleosome density was examined across the gene. For the most part, the density was evenly distributed in cells that were not expressing *HLA-DRA*. However, when *HLA-DRA* was expressed, there was a notable decrease in the nucleosome density immediately upstream of the TSS. This decrease was not seen in either the CIITA- or RFX5-deficient cell lines or in control fibroblasts. This suggests that CIITA recruitment was necessary for altering the nucleosome density, and as discussed below, many of the other chromatin modulating events at the locus. The decrease in nucleosome density may maintain an accessible chromatin environment close to the TSS to allow efficient assembly of RNA polymerase components and subsequent transcription initiation.

With activation of transcription, histone modifications associated with active gene expression were present to varying degrees and patterns across the *HLA-DRA* locus. While the distribution patterns of each mark were largely similar between the IFN-γ induced cells and B cells, minor differences were observed. The difference in ChIP assay levels in general may reflect the difference between newly applied histone modifications as in the case of IFN-γ treated A431 cells, and constitutive levels of the same histone modifications in the B cells. The levels of histone modification observed in IFN-γ treated cells imply that histone modifying factors were newly recruited, whereas in both wild-type and mutant B cell lines, regulatory factors had the opportunity to establish a steady state of modifications by constant new application and removal of the marks. The dissimilarities of modification patterns were mostly at the *XL4* region, where higher levels of several modifications were observed in B cells compared to IFN-γ induced cells. H4K8ac was an exception to this as it was higher at *XL4* in the IFN-γ induced cells. These observations may reflect the potential for differential use of *XL4* as a regulatory element in the different cell types and conditions. The data collected here suggest that *XL4* is likely more important for B-cell specific than for the IFN-γ induced expression. Alternatively, the use of *XL4* may correlate with higher levels of *HLA-DRA* transcription in Raji cells.

The three histone H3K4 methylation modifications had markedly distinct distribution patterns across the *HLA-DRA* gene. H3K4me3 was highly enriched in a focused region surrounding and just downstream of the TSS in both IFN-γ induced cells and B cells. This is an expected result as most active genes have a biphasic peak surrounding the TSS [55]. Although diminished significantly, H3K4me3 was present in the CIITA-deficient cell line but not the RFX5-deficient line. Because the RFX-CREB-NF-Y ternary complex is assembled in RJ2.2.5 cells [56], the data suggest that these factors can at some level recruit the necessary KMT to write this mark close to the TSS. A similar observation was made for H3K4me2 as well. The H3K4me1 modification was highest in both CIITA- and RFX5-deficient cells, indicating that the factors bound in these cells are capable of recruiting the KMT responsible for this modification. Thus, in the most basal state, H3K4me1 is placed at the TSS region, and the assembly of the RFX-CREB-NF-Y factors and subsequent CIITA recruitment results in additional methylation of H3K4. The finding of any modifications in SJO (RFX5-deficient) was unexpected as previous reports had shown that in the absence of a functional RFX, the other DNA binding factors (CREB and NF-Y) do not assemble [57]. However, the finding of the histone modifications in SJO cells could represent transient binding of NF-Y or CREB to these sites with the subsequent recruitment of KAT/KMTs and establishment of the observed histone modifications.

Intriguingly, in the *HLA-DRA* expressing B cells, there was an enrichment of H3K4me1 at +1500 similar to the levels observed at *XL4* in all three B cell lines. While this mark is known to be present within the body of genes [14], the +1500 did have significantly more of this mark than surrounding areas, suggesting the possibility of a novel regulatory region within the vicinity. Twenty-four years ago, a paper reported that this region of the *HLA-DRA* intron had tissue specific enhancer activity [58]. This modification may reflect that activity. It is intriguing that the modification was not observed in the CIITA- or RFX5-deficient cells, suggesting that it is CIITA dependent. Similarly, in the IFN-γ induced fibroblasts H3K4me1 was enriched at −600 compared to the other sequences. This region has no known function at this time, but like the +1500 region, could represent a novel control element for this gene.

The addition of a secondary protein crosslinker to the standard ChIP assay allowed the identification and demonstration of the clear association of coactivators associated with the *HLA-DRA* gene. The KATs CBP, p300, GCN5, and PCAF have been previously described to associate with CIITA in cell lysates [15], [22], [23], and CBP and GCN5 have been shown by ChIP to be bound to *HLA-DRA* WXY box regions in *MHC-II* expressing cells [30], [31]. Here, each of these factors was shown to be recruited to the *HLA-DRA* WXY box region in a CIITA- and RFX5-dependent manner. Of the histone acetylation modifications observed, H3K9ac and H3K18ac were the most prominent, and these marks have been strongly associated with the KAT activities of GCN5/PCAF and CBP/p300, respectively [59], [60], [61], [62], and are likely responsible for placing these marks on *HLA-DRA*.

In B cells, there were significantly higher levels of enrichment within the body of the gene for GCN5 and PCAF, when compared to CBP and p300. GCN5 has been shown to bind within the body of genes and has been implicated in transcriptional elongation [63], [64]. Inducible genes can be heavily regulated at the elongation step and GCN5 may be regulating transcription by acting as a gatekeeper for elongation under the appropriate signals. GCN5 and PCAF are also capable of acetylating transcription factors. PCAF was shown to acetylate CIITA and regulate its nuclear localization [23]. These reports support the notion that while these KATs are globally recruited to actively transcribed genes, they use different and/or multiple mechanisms to activate genes.

GCN5, the first KAT identified to be involved in transcriptional activation, functions as part of the well-characterized yeast SAGA complex [65]. In vertebrates PCAF shares 75% homology with GCN5 and both have somewhat redundant functions [66]. Recent work in metazoans has further divided the SAGA complexes into STAGA and ATAC, which share a common core of GCN5/PCAF, ADA3, STAF36, and a homolog of the yeast ADA2, being either ADA2a in ATAC complexes, or ADA2b in STAGA complexes [53]. STAGA and ATAC contain other unique subunits that allow them to function at separate target genes largely in a mutually exclusive manner [67]. While the results presented here showed enrichment for ADA2a, a component of the ATAC complex, but not ADA2b of the STAGA complex at *HLA-DRA* promoter and *XL4*, other components of the STAGA complex were present. The results presented here suggest other complexes for GCN5/PCAF may exist or that the components assemble independently of the larger complexes depending on the unique activating transcription factors available at each gene.

In sharp contrast to the other coactivators, the binding of WDR5 in B cells was completely independent of CIITA and RFX5. This suggests that WDR5 may be recruited to the region by the WXY box factors prior to CIITA and acts as a docking site for its partner histone modifying proteins. Although the other MLL complex proteins, MLL1, ASH2L, RbBP5, and DPY-30 bound to varying levels in the absence of CIITA, their occupancy increased when CIITA was bound. This suggests that WDR5 is recruited through multiple mechanisms and potentially multiple complexes. WDR5 is reported to be present in both the MLL and ATAC complexes [47], [53]. A recent publication showed the NF-Y complex was able to recruit ASH2L to CCAAT containing promoters [68]. As NF-Y proteins are also part of the MHC-II promoter-binding complex, this could partially explain the CIITA-independent binding of MLL complex components. Also, a WDR5, ASH2L, and RbBP5 complex was shown to catalyze H3K4 mono- and di- methylation activity independent of MLL [69]. By applying a basal level of H3K4 methylation independent of CIITA, these proteins may function to increase the accessibility of the chromatin at these sites, which can be modified further with the recruitment of MLL and other coactivators.

siRNA knockdown of GCN5, MLL1, and WDR5 could not provide evidence that these factors were essential to *HLA-DRA* gene expression. While the data could be interpreted that the components are not required or are redundant, another interpretation is also possible. This possibility involves the programming of this gene and the epigenetic stability of the histone modifications that were placed following activation. When IFN-γ was removed from the inducible system, the level of *HLA-DRA* was unaltered despite the loss of *CIITA* transcription. This may be partly due to the stability of the *HLA-DRA* mRNA itself. However, the data suggest that this is more likely a consequence of a number of events, including the presence of active histone modifications that are stably associated with the promoter. Albeit reduced, CIITA was also readily detectable at three and five days post IFN-γ removal from the system. Thus, there is an inherent stability of CIITA binding and the histone modifications associated with the locus once it is activated.

In summary, the results presented here illustrate the complexity in activating the *HLA-DRA* gene and showed that multiple KATs and KMTs are involved in the process. For histone acetylation, it is clear that CIITA is critically required for the placement of these marks. Even with this being the case, some of the factors can be recruited to the gene in the absence of CIITA, implying that their recruitment may not in its self be sufficient for them to catalyze these modifications. For histone methylation, CIITA-dependent and independent recruitment of factors occurs. However, some of these factors are active in the absence of CIITA, creating a state in which this region is open and accessible. Thus, their role may be to maintain a constitutively accessible state, such that these important adaptive immune response set of genes can be induced rapidly in response to infections.

## Supporting Information

Figure S1 **IFN-γ treatment induced the deposition of active histone modifications throughout the** ***HLA-DRA*** **gene, plotted with respect to histone H3 density.** The data from Figure 1C were replotted as fold over the histone H3 percent of input chromatin values for each amplicon as determined by histone H3 ChIP.
(TIF)

Figure S2 ***MHC-II*** **expressing B cells have active histone modifications distributed across the** ***HLA-DRA*** **gene, plotted with respect to histone H3 density.** The data from Figure 2B were replotted as fold over the histone H3 percent of input chromatin values for each amplicon as determined by histone H3 ChIP**.**
(TIF)

Figure S3 **Histone modifying proteins in Raji, RJ2.2.5, and SJO cells are expressed at similar levels.** Nuclear extracts from Raji, RJ2.2.5, and SJO cells were prepared and equally loaded on SDS-PAGE, blotted to PVDF membranes and stained with the indicated antibodies as described in materials and methods. Molecular weight (MW) are shown.
(TIF)

Figure S4 **GCN5 complex component ChIP from IFN-γ treated A431 cells.** Dual crosslinking ChIP was performed on A431 cells −/+ IFN-γ for 24 hours as described in the text of the manuscript. These data represent the average of three biological replicates. Asterisks represent data values that were statistically significant (Student’s t-test p<0.05) when compared to the IgG control ChIP assays.
(TIF)
