# Supplementary figures and images for: Multiple Histone Methyl and Acetyltransferase Complex Components Bind the HLA-DRA Gene
Source: PLoS One. 2012 May 31;7(5):e37554. doi: 10.1371/journal.pone.0037554 (PMC3365104; doi:10.1371/journal.pone.0037554)

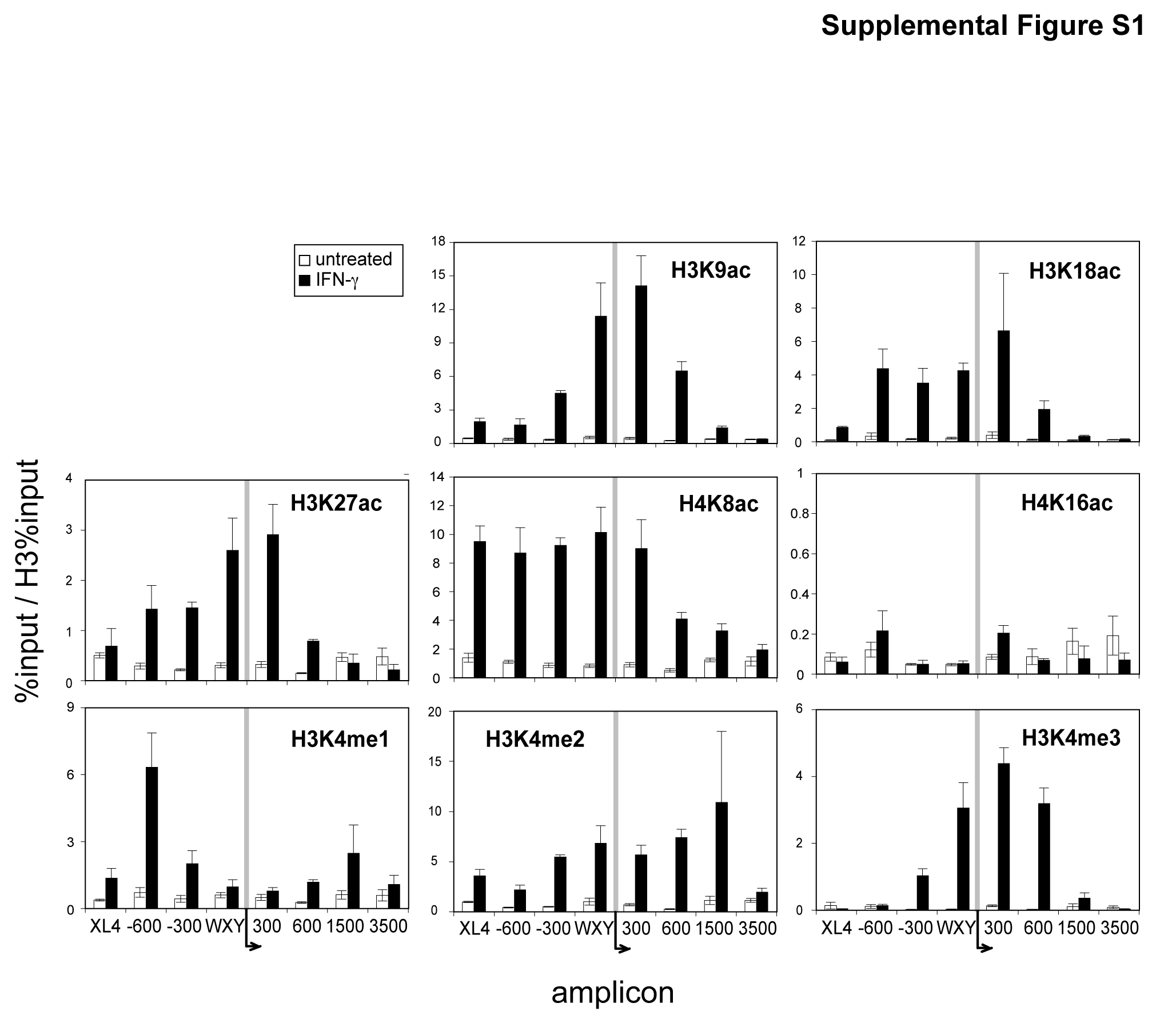

Supplement: Figure S1 — IFN-γ treatment induced the deposition of active histone modifications throughout the HLA-DRA gene, plotted with respect to histone H3 density. The data from Figure 1C were replotted as fold over the histone H3 percent of input chromatin values for each amplicon as determined by histone H3 ChIP. (TIF) [file pone.0037554.s001.tif]

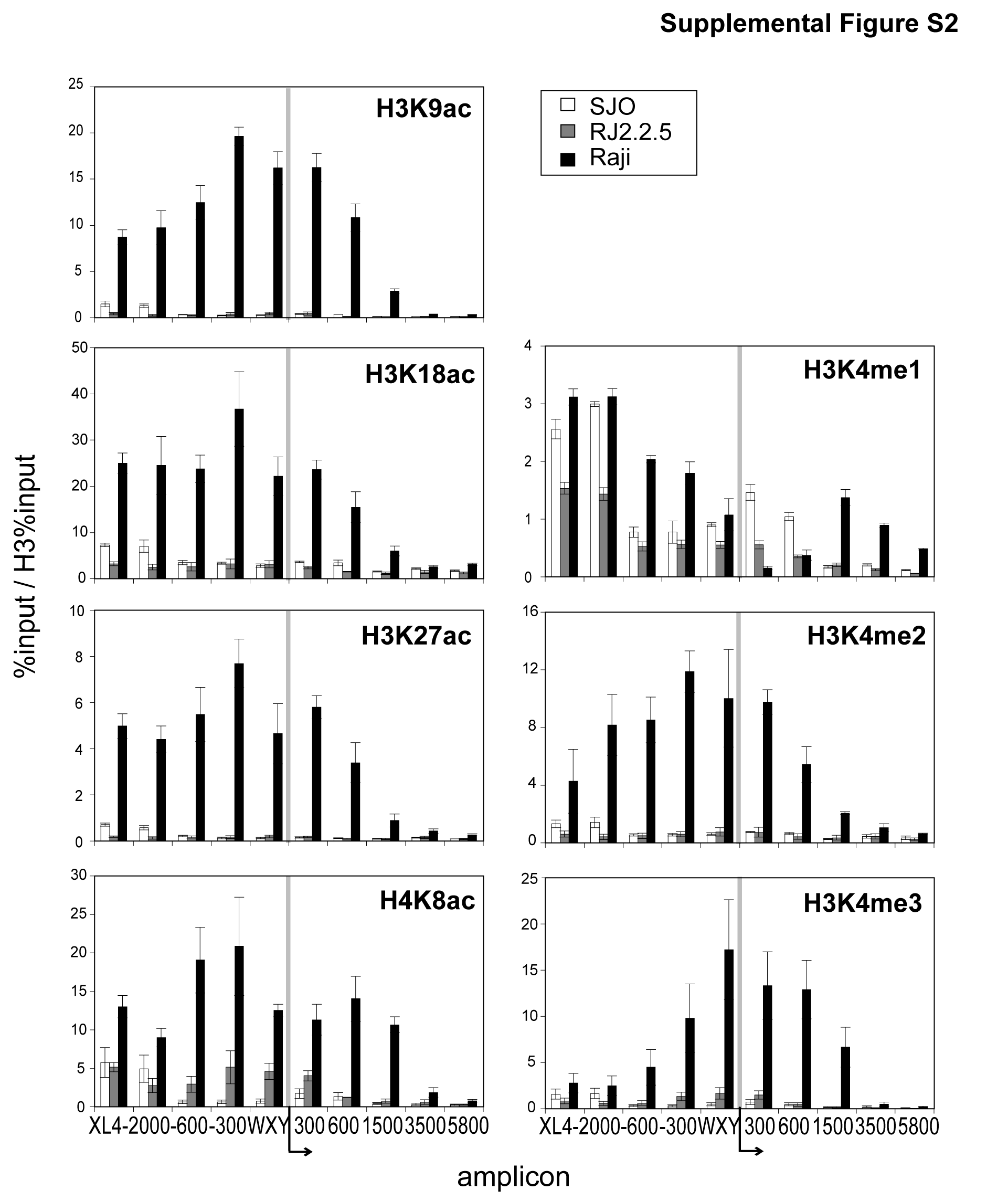

Supplement: Figure S2 — MHC-II expressing B cells have active histone modifications distributed across the HLA-DRA gene, plotted with respect to histone H3 density. The data from Figure 2B were replotted as fold over the histone H3 percent of input chromatin values for each amplicon as determined by histone H3 ChIP. (TIF) [file pone.0037554.s002.tif]

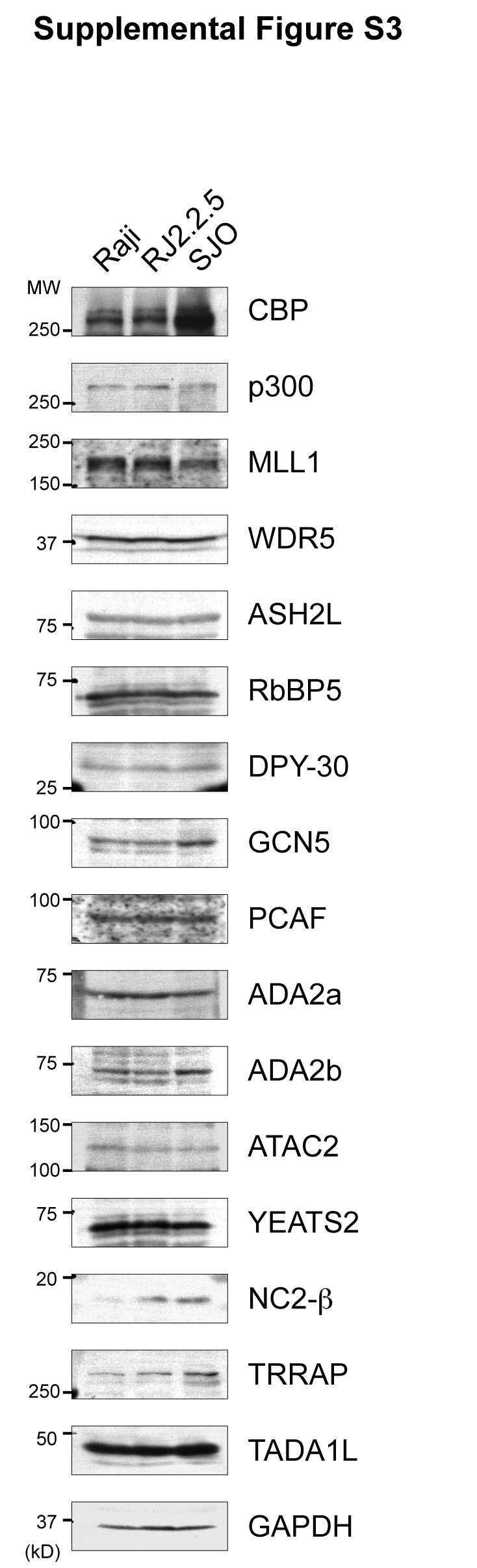

Supplement: Figure S3 — Histone modifying proteins in Raji, RJ2.2.5, and SJO cells are expressed at similar levels. Nuclear extracts from Raji, RJ2.2.5, and SJO cells were prepared and equally loaded on SDS-PAGE, blotted to PVDF membranes and stained with the indicated antibodies as described in materials and methods. Molecular weight (MW) are shown. (TIF) [file pone.0037554.s003.tif]

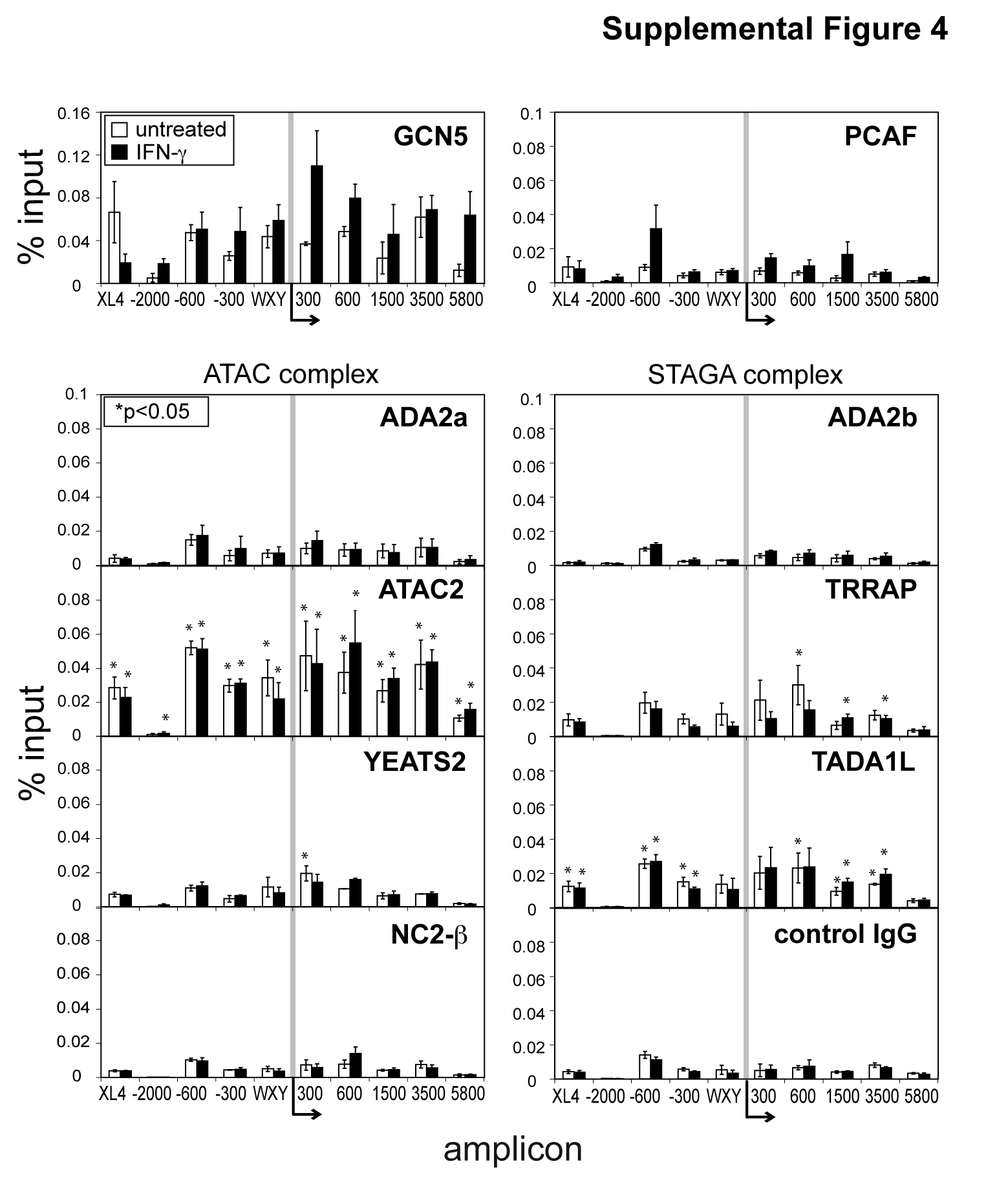

Supplement: Figure S4 — GCN5 complex component ChIP from IFN-γ treated A431 cells. Dual crosslinking ChIP was performed on A431 cells −/+ IFN-γ for 24 hours as described in the text of the manuscript. These data represent the average of three biological replicates. Asterisks represent data values that were statistically significant (Student’s t-test p<0.05) when compared to the IgG control ChIP assays. (TIF) [file pone.0037554.s004.tif]
